# Evaluation of immunity against malaria using luciferase-expressing *Plasmodium berghei *parasites

**DOI:** 10.1186/1475-2875-10-350

**Published:** 2011-12-09

**Authors:** Ivo Ploemen, Marije Behet, Krystelle Nganou-Makamdop, Geert-Jan van Gemert, Else Bijker, Cornelus Hermsen, Robert Sauerwein

**Affiliations:** 1 Department of Medical Microbiology, Radboud University Nijmegen Medical Center (RUNMC), Nijmegen, The Netherlands

## Abstract

**Background:**

Measurement of liver stage development is of key interest in malaria biology and vaccine studies. Parasite development in liver cells can be visualized in real-time, both in culture and in live mice, using a transgenic *Plasmodium berghei *parasite, *Pb*GFP-Luc_con_, expressing the bioluminescent reporter luciferase. This study explores the benefit of using these parasites for the evaluation of immunity against malaria, compared to qRT-PCR techniques *in vivo *and *in vitro*.

**Methods:**

Mice were immunized with either radiation attenuated sporozoites (RAS) or wildtype sporozoites under chloroquine prophylaxis (CPS) and challenged with *Pb*GFP-Luc_con. _The *in vitro *transgenic sporozoites neutralization assay (TSNA) was adapted by replacing *Pb*CS(Pf) parasites for *Pb*GFP-Luc_con _parasites.

**Results:**

Application of *Pb*GFP-Luc_con _transgenic parasites provides live quantitative visual information about the relation between parasite liver load and protection. Moreover, fast and reproducible results are obtained by using these parasites in the transgenic sporozoites neutralization assay, measuring functional antibody-mediated immune responses.

**Conclusions:**

*Pb*GFP-Luc_con _parasites are a straightforward and valuable tool for comprehension of the biological and immunological principles underlying protection against malaria.

## Background

Transgenic organisms that express a bioluminescent reporter are increasingly used due to easy handling and visualization. *Plasmodium berghei *parasites, expressing the bioluminescent reporter luciferase (*Pb*GFP-Luc_con_) have been used to visualize and quantify parasite development *in vitro *in hepatic cells and *in vivo *in mice using real-time luminescence imaging [1].

Measurement of liver stage development is of key interest in malaria biology and vaccine studies. Protection against the liver stage is one of the targets to abrogate the infection. Quantification of the number of parasites in hepatocytes is an important read-out to determine inhibitory activity. This quantification of *in vitro *[2] and *in vivo *[3] parasite liver load is usually performed by (qRT)-PCR. This technique, however, is time-consuming and costly, since mice need to be sacrificed at each time point for *in vitro *quantification.

The use of *in vivo *and *in vitro *imaging of luciferase expressing parasites has some requisites. First, it requires that the luciferase expressing parasites are qualitative and quantitative biologically comparable to wildtype in terms of liver and blood infectivity. Second, the *in vivo *and *in vitro *parasite quantification by measurement of luminescence signaling needs to correlate to the established qRT-PCR methods. Previously we showed that the *P. berghei *line 676m1cl1 line (*Pb*GFP-Luc_con_) and wildtype (WT) sporozoites have identical motility, cell traversal and *in vitro *and *in vivo *hepatocyte infectivity. Moreover, detailed examination revealed that luciferase expression correlated tightly with parasite 18S rRNA levels measured by qRT-PCR [1]. Therefore, this transgenic parasite seems suitable for a quantitative analysis of parasite load.

This study aimed to explore the use of *Pb*GFP-Luc_con _parasites in both *in vivo *and *in vitro *studies evaluating immunity against malaria. For the *in vivo *studies, mice were immunized with either radiation attenuated sporozoites (RAS) or wildtype sporozoites under chloroquine prophylaxis (CPS) and subsequently challenged with *Pb*GFP-Luc_con_. For the *in vitro *studies, the transgenic sporozoites neutralization assay (TSNA) was adapted by replacing *Pb*CS(Pf) parasites for *Pb*GFP-Luc_con _parasites [2].

## Methods

### Mice

Female C57BL6/J mice, eight weeks of age, were purchased from Elevage Janvier (France). All studies have been performed according to the regulations of the Dutch "Animal On Experimentation act" and the European guidelines 86/609/EEG. Approval was obtained from the Radboud University Experimental Animal Ethical Committee (RUDEC 2009-019).

### Mosquito infection and preparation of sporozoites

The previously described, transgenic *P. berghei *line 676m1cl1 line (*Pb*GFP-Luc_con_) [1] and its reference clone of ANKA strain cl15cy1, were used in this study.

*Anopheles stephensi *mosquitoes were infected by feeding on infected mice using standard methods of mosquito infection [4]. On day 21 after infection, the salivary glands of the mosquitoes were collected by hand-dissection. Salivary glands were collected in DMEM (Dulbecco's Modified Eagle Medium from GIBCO) and homogenized in a homemade glass grinder. The free sporozoites were counted in a Bürker-Türk counting chamber using phase-contrast microscopy.

### Immunization of mice with radiation attenuated sporozoites (RAS) or sporozoites under chloroquine prophylaxis (CPS)

C57BL/6 mice were immunized with wildtype *P. berghei *radiation attenuated sporozoites (RAS) or sporozoites under chloroquine prophylaxis (CPS). Immunizations were performed by i.v injection with three doses of 1 × 10^4 ^(RAS and CPS) or 4 × 10^3 ^(CPS) sporozoites, with a 7 day interval between the boosts. For CPS immunization, mice received 800 μg chloroquine base (cq-diphosphate Sigma) in PBS i.p, starting from sporozoite injection up to two weeks after the last immunization. Absence of blood stage parasites was confirmed by examination of Giemsa-stained blood smears of tail blood at the end of the chloroquine treatment period and approximately 1 day before challenge. Mice were challenged two weeks after ending choroquine treatment. Irradiation of sporozoites was performed by exposure of infected *A. stephensi *mosquitoes to 16,000 rad of γ-radiation (Cesium-137 Gammacel 1000).

### Challenge and real time *in vivo *imaging of liver stage development in RAS and CPS immunized mice

Immunized and control C57BL/6 mice were challenged by the bite of 5-11 infectious mosquitoes or by intravenous injection of 1 × 10^4 ^*Pb*GFP-Luc_con _sporozoites in the tail (200 ul). Control mice consisted of two groups, group 1 received chloroquine similar to the CPS immunized mice and group 2 did not receive chloroquine. Giemsa stained bloodsmears were prepared every other day starting from day 3 to day 21 after challenge, to monitor for blood stage parasitaemia. Parasite liver load in animals was visualized through imaging of whole bodies using the *in vivo *imaging system Lumina (Caliper Life Sciences, USA) as described [1], with some small adaptations. Briefly, animals were anesthetized using the isofluorane-anesthesia system, their belly was shaved and D-luciferin dissolved in PBS (100 mg/kg; Caliper Life Science, Belgium) was injected subcutaneously (in the neck). Animals were kept anesthetized during the measurements, performed within 3-5 minutes after the injection of D-luciferin. Bioluminescence imaging was performed with a 10 cm field of view, medium binning factor and an exposure time of 300 seconds. Bioluminescent intensities were expressed in total flux of photons.

### Real time transgenic PbGFP-Luc_con _sporozoites neutralization assay

**Figure 1:**
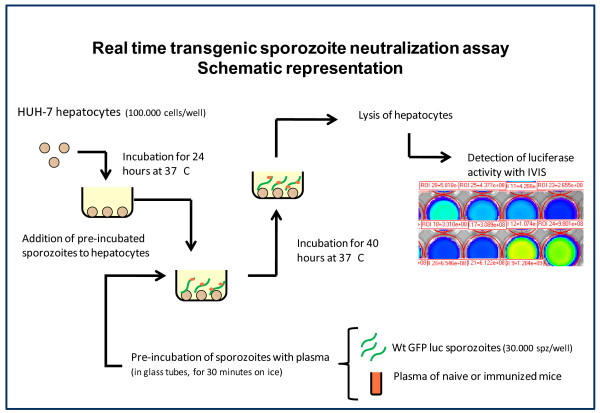
**Schematic representation of the adapted transgenic *Pb*GFP-Luc_*con *_sporozoite neutralization assay**. Neutralization of hepatocyte invasion by transgenic sporozoites was performed by incubation of naive or immune plasma obtained from (non-) immunized mice with the transgenic sporozoites. Neutralization was performed for 30 minutes on ice before the antibody/sporozoites mix was added to Huh-7 cells containing wells and incubated for 40 h at 37°C. This figure is adapted from figure 1 described by Kumar *et al. *[2]

The TSNA (transgenic sporozoites neutralization assay) protocol was adapted from Kumar *et al. *(Figure 1). Plasma was obtained from immunized (three doses of 1 × 10^4 ^;RAS and CPS) and naive C57BL/6 mice, 21 days post challenge by mosquito bite; blood was collected by heart puncture after i.v. injection of 50 i.u. of heparin. Blood samples were centrifuged at 2000 rpm for 5 minutes (RT), plasma was collected and transferred to cryotubes (Nunc) and stored at -80°C for later use. Prior to the TSNA assay, plasma samples were thawed and centrifuged at 13,000 rpm for one minute (RT) to remove protein aggregates. *Pb*GFP-Luc_*con *_sporozoites were pre-incubated for 30 minutes on ice with plasma of naive or immunized mice (1:1 ratio).

Pre-incubated sporozoites were added to wells containing monolayers of 1 × 10^5 ^pre-seeded Huh-7 hepatocyte cultures (1 ml/well in 24 well plates). Huh-7 cells (human liver hepatoma cells) were preferred over standard HepG2 cells [2] since in these cells, luciferase expression correlated slightly better with parasite 18S rRNA levels measured by qRT-PCR [1]. Huh-7 were suspended in 1 ml of 'complete' DMEM (DMEM, Gibco, supplemented with 10% FCS, 1% penicillin/streptomycin and 1% Glutamax) the day prior to infection, seeded in 24 well plates (10^5 ^cells/well) and incubated overnight. For each plasma sample, 3 × 10^4 ^sporozoites each were added to duplicate wells and plates were centrifuged 10 minutes at 1800 × g (Eppendorf centrifuge 5810 R). 40 hours after sporozoite addition, cells were washed once with PBS and lysed in 200 μl of cell culture lysis reagent obtained from the Promega Luciferase Assay System Kit^® ^(Promega, PT). Samples in Promega lysis buffer were either stored at -80°C or processed immediately to measure luminescence intensity with the Lumina system. The *in vivo *imaging system Lumina (Caliper Life Sciences, USA) was used to measure luciferase activity of infected Huh-7 cells. Quantitative analysis was performed by measuring the luminescence signal intensity per well using the ROI settings of the Living Image^® ^3.0 software. ROI measurements are expressed in total flux of photons. 70 μl of Luciferase Assay Substrate (Promega Luciferase Assay System Kit^®^) was added to 20 μl of lysed hepatocyte cultures in a white 96-well plate (Dynex Technologies, USA). Bioluminescence images were acquired with a 7 cm FOV, medium binning factor and an exposure time of 10-30 seconds. Percent inhibition was calculated by the following formula; 1 - (average bioluminescence in immune plasma cultures/average bioluminescence in naive plasma cultures) × 100%.

## Results

### Challenge of immunized mice with PbGFP-Luc_con _sporozoites

**Figure 2:**
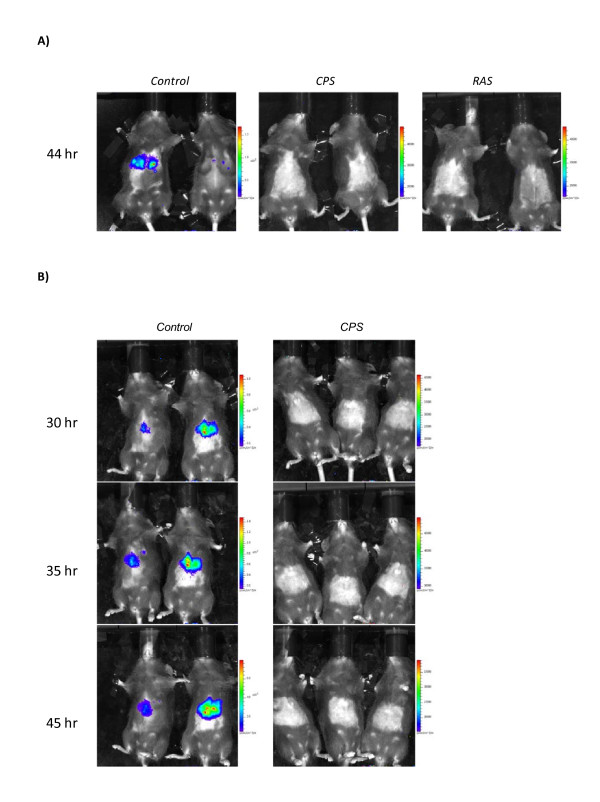
**Real-time *in vivo *parasite liver load upon challenge in mice immunized with CPS or RAS**. (A) Image (2 representative mice for each group) of the parasite liver load in control (n = 10), CPS (n = 10) and RAS immunized (n = 10) C57BL/6 mice 44 hours post challenge. Mice were immunized i.v with 1 × 10^4 ^sporozoites followed by two boosts of 1 × 10^4 ^sporozoites. Challenge was performed by infectious mosquito bites. The rainbow image visible in the naive mice represents the total flux of photons (p/sec/cm^2^) in that area. (B) Image (2 control mice and 3 immunized mice) of the parasite liver load in control (n = 3) and CPS immunized (n = 5) C57BL/6 mice 30-45 hours post challenge. Mice were immunized with 4 × 10^3 ^sporozoites by i.v injection followed by two boosts of 4 × 10^3 ^sporozoites. Challenge was performed by injection of 1 × 10^4 ^*Pb*GFP-Luc_*con *_sporozoites i.v. The rainbow image visible in the control mice represents the total flux of photons (p/sec/cm^2^) in that area.

Mice immunized with CPS or RAS as well as control mice were challenged by *Pb*GFP-Luc_con _infected mosquitoes and protection against malaria was evaluated by blood smear reading and real time *in vivo *imaging. All control challenged mice (n = 10) developed asexual parasitaemia and a positive bioluminescent liver signal by real-time *in vivo *imaging at 44 hours post challenge (Figure 2a). All mice immunized by CPS (n = 10) or RAS (n = 10) with a dose regimen of three times 10^4 ^sporozoites and challenged by infectious mosquito bites, neither became parasitaemic nor displayed any bioluminescent signal originating from the liver (Figure 2a). Next, the robustness of protective immunity was explored by increasing the challenge level in mice that were immunized with CPS by a lower dose regimen of three times 4 × 10^3 ^sporozoites. Mice were challenged by i.v injection of 1 × 10^4 ^*Pb*GFP-Luc_con _sporozoites and all immunized mice remained negative. These results are in line with the data obtained by *in vivo *imaging; mice immunized with CPS showed no bioluminescent signal, in contrast to control mice, with positive images at 30 to 45 hours post challenge (Figure 2b). Therefore, the use of *Pb*GFP-Luc_con _in a challenge model, combined with bioluminescent imaging permits determination of protective efficacy in the liver post-immunization.

### Real time transgenic PbGFP-Luc_con _sporozoites neutralization assay

**Figure 3:**
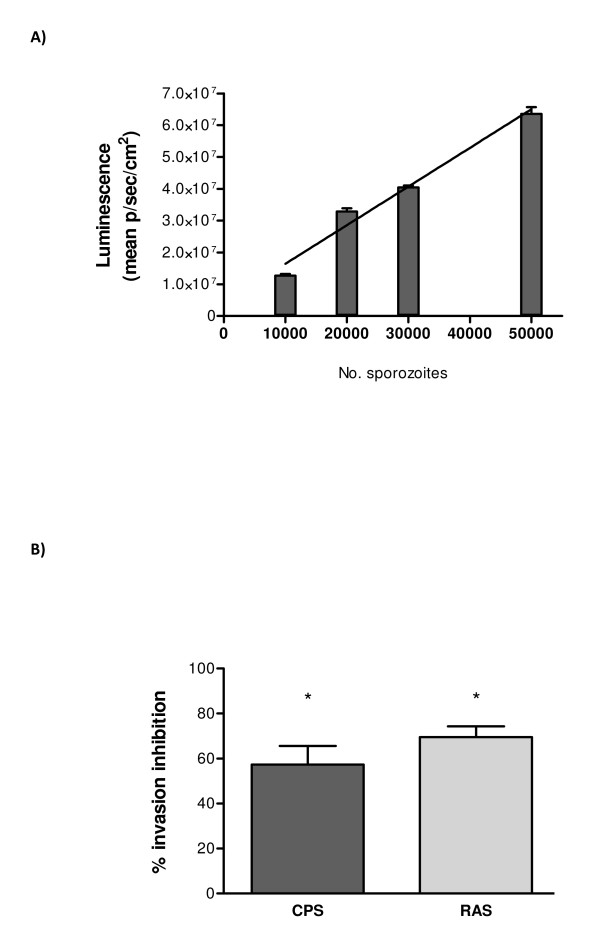
**Transgenic *Pb*GFP-Luc_*con *_sporozoite hepatocyte invasion inhibition assay**. (A) Huh-7 cells were seeded in 24 well plates and were grown to confluency as described in Materials and Methods. Graded numbers of *Pb*GFP-Luc_*con *_sporozoites were added to the culture wells in duplicate and incubated for 40 h at 37 C in 5% CO_2_. Infectivity was quantified by analyzing the luminescent flux (p/sec/cm^2^) in each well. (B) Invasion inhibition of sporozoites by plasma from CPS (n = 6) and RAS (n = 7) immunized mice. 30.000 *Pb*GFP-Luc_*con *_sporozoites were incubated in plasma of naive or immune mice for 30 min on ice and subsequently added to Huh-7 cells. Infectivity was quantified by analyzing the luminescent flux (p/sec/cm^2^) in each well. The baseline represents the inhibition level of plasma from naive mice (approximately 4 × 10^7 p/sec/cm^2^). Percent inhibition was calculated by the following formula; 1 - (average bioluminescence in immune plasma sample/average bioluminescence in naive plasma sample) × 100%. The level of invasion inhibition in the immunized mice was significantly higher compared to the inhibition from the plasma of naive mice (95% CI CPS 36-79%; 95% CI RAS 58-81%).

To evaluate the potential benefits of *Pb*GFP-Luc_con _for assessment of protection *in vitro*, we adapted the transgenic sporozoite invasion inhibition assay (TSNA) as performed by Kumar *et al. *[2] by replacing the *Pb*CS(Pf) sporozoites with *Pb*GFP-Luc_con _sporozoites. The use of bioluminescent parasites in the TSNA has some requisites. Recently, Ploemen *et al. *described the highly significant quantitative correlation between parasite 18S rRNA levels measured by qRT-PCR and luminescence intensity for different numbers of *Pb*GFP-Luc_con _sporozoites invaded into Huh-7 hepatocytes (Spearman rho = 0.83) [1]. As a follow-up of these results, a dose titration with 10 - 50.000 *Pb*GFP-Luc_con _sporozoites, added to a Huh-7 hepatocyte culture was performed and bioluminescent intensity was measured 40 hrs post invasion (Figure 3a). Luminescent intensities increased linear (R^2 ^= 0.97) with the number of *Pb*GFP-Luc_*con *_sporozoites added to a Huh-7 hepatocyte culture. These parasites, therefore, may be used in an adapted TSNA.

Plasma from protected C57BL/6 mice was used in the adapted TSNA, after CPS or RAS immunization. Invasion of Huh-7 cells by *Pb*GFP-Luc_con _sporozoites pre-incubated with plasma of protected mice was significantly inhibited compared to invasion by sporozoites pre-incubated with plasma of naive mice (p < 0.05) (Figure 3b). Further, 1:1 diluted plasma (in PBS), showed about half of the inhibition level of the original plasma (data not shown). Inhibition of sporozoites by purified plasma IgG of protected mice resulted in a similar inhibition level as non-purified plasma (data not shown). Although the % inhibition between individual plasma samples differs between mice, the duplicates of one plasma sample diverged at most on average 4% of the mean value of that sample (n = 21). These results show that this adapted TSNA is a more user friendly methodology, allowing identification of antibody-mediated inhibition of parasite liver invasion.

## Discussion

This study shows that *Pb*GFP-Luc_con _parasites, can provide real-time quantitative information on the relation between *in vivo *parasite liver load and immunity against malaria. *In vitro *use of these parasites in the adapted TSNA allows for an easy and fast assessment of the functional sporozoite invasion inhibition by antibodies.

Previously, Mwakingwe *et al. *applied bioluminescent imaging and qRT-PCR to analyse parasite prevalence in the liver of immunized mice, using luciferase expressing *P. yoelii *[5]. They did not, however, report on the direct quantitative relation between bioluminescent imaging and parasite (liver) load determined by qRT-PCR and the extent to which the transgenic parasite had similar characteristics as the WT parasite throughout the whole life cycle [5]. In the future, a *Py-*Luc parasite that meets these requisites for assessment of the liver load upon challenge, can be used beside *Pb*GFP-Luc_con _parasites, allowing for a direct comparison of the characteristics of *P. yoelii *and *P. berghei in vivo*.

Evaluation of immunity against malaria by bioluminescent imaging offers many advantages over conventional qRT-PCR analysis. This analysis technique is more simple, rapid and reduces the amount of mice needed. As an added value, expression of the reporter protein luciferase is restricted to live parasites and therefore allows specific detection of live parasites. This avoids detection of dead liver parasites, as may occur by the qRT-PCR assay [1]. Measurement of parasite liver load upon challenge can be performed *in vivo *without the need for any invasive liver resection or biopsy. It does not require sacrificing animals and thereby reduces the number of animals and costs required for experimentation. Moreover, multiple measurements can be carried out in the same animal over time, linking the parasite liver load with protection and minimizing the effect of biological variation [6,7]. While the use of *Pb*GFP-Luc_con _over qRT-PCR has its clear benefits, there are limitations. The expression of luciferase in the parasite is relatively low and cannot be visualized earlier than 20 hours post-infection [1]. Moreover, due to limitations in sensitivity, a low number of developing liver parasites may be missed which might mature into asexual parasites [1]. Negative results of *in vivo *imaging of liver stage development can therefore not be used to claim sterile protection, which eventually requires sub-inoculation of blood from challenged mice into naive mice [3]. Nonetheless, challenge of mice with a high number of *Pb*GFP-Luc_con _sporozoites administered i.v does offer information on the parasite liver load in real time without sacrifying the mice. In the CPS model the overwhelming majority of the parasites do not develop in the liver beyond 30 hours. The presence of effector mechanisms that target early developing parasite stages can however, not formally be excluded. At least in this model with complete liver stage development, a high degree of immunity to late liver stage parasites can clearly be inferred.

Finally, the application of *Pb*GFP-Luc_con _parasites is not restricted to the described assays. In a recent publication describing the host mediated factors regulating the inhibition of liver stage infection upon superinfection, luciferase expressing parasites were used to enable distinction between the parasites from the original infection and the superinfection [8].

## Conclusions

With clear benefits over conventional RT-qPCR techniques, *Pb*GFP-Luc_con _parasites can function as an easy and valuable tool contributing to the comprehension of the immunological principles underlying immunity against malaria. As such, these parasites can be helpful in future studies evaluating protection against malaria.

## List of abbreviations

*Pb*GFP-Luc_con_: *Plasmodium berghei *that constitutively express firefly Luciferase and the Green fluorescent protein; *Pb*CS(pf): *Plasmodium berghei *that bears the *Plasmodium falciparum *CS repeats; TSNA: Transgenic sporozoite neutralization assay; RAS: Radiation attenuated sporozoites; CPS: Sporozoites under chloroquine prophylaxis; ROI: Region of interest; q-RTPCR: quantitative real-time polymerase chain reaction; IVIS: in vivo imaging system.

## Competing interests

The authors declare that they have no competing interests.

## Authors' contributions

IP conceived the study, which was largely carried out by IP and MB. KN, GvG and EB, helped design the studies and carry out immunizations and challenge. IP, MB and CH discussed experiments and results. IP wrote the manuscript which was edited by CH and RS. All authors read and approved the final manuscript.
